# Update on surgical management of complex macular holes: a review

**DOI:** 10.1186/s40942-021-00350-4

**Published:** 2021-12-20

**Authors:** Mohd-Asyraaf Abdul-Kadir, Lik Thai Lim

**Affiliations:** Present Address: Department of Ophthalmology, Universiti Malaysia Sarawak (UNIMAS), Kota Samarahan, Malaysia

**Keywords:** Macular hole, Vitrectomy, Vitreoretinal disease, Internal limiting membrane peeling

## Abstract

Modern surgical interventions effectively treat macular holes (MHs) more than 90%. Current surgical treatment for MHs is pars plana vitrectomy with epiretinal membrane, internal limiting membrane (ILM) peeling, gas endotamponade, and prone posturing postoperatively. However, a small subset of MHs imposes challenges to surgeons and frustrations on patients. A narrative review was performed on the surgical treatment of challenging MHs including large and extra-large MHs, myopic MHs with or without retinal detachment, and chronic and refractory MHs. There are robust data supporting inverted ILM flap as the first-line treatment for large idiopathic MHs and certain secondary MHs including myopic MHs. In addition, several studies had shown that ILM flap manipulations in combination with surgical adjuncts increase surgical success, especially in difficult MHs. Even in eyes with limited ILM, surgical options included autologous retinal graft, human amniotic membrane, and creation of a distal ILM flap that can assist in MH closure even though the functional outcome may be affected by the MH chronicity. Despite relative success anatomically and visually after each technique, most techniques require a long-term study to analyze their safety profile and to establish any morphological changes of the MH plug in the closed MHs.

## Introduction

Macular holes (MHs) were once considered non-treatable and an MH was first described in 1869 from a traumatic origin [1]. MH is characterized by a vertical defect in the neurosensory retinal anatomy particularly in the foveal region that extends from the internal limiting membrane (ILM) to the retinal pigment epithelium (RPE) and it affects the central vision and causes metamorphopsia [2]. MHs are predominantly idiopathic (primary) with higher prevalence with increasing age and in females. Its estimated annual incidence is up to 8.69 eyes in 100, 000 population [3, 4]. Secondary MHs are attributed to but not limited to high myopia, trauma, proliferative diabetic retinopathy, and various retinal pathologies.

Current surgical techniques successfully close majority MHs greater than 90% with remarkable visual acuity gain; however, small percentages of MHs have a higher risk of initial surgical failure [5, 6]. Large MHs, MHs with a basal diameter of > 400 μm, are likely to have a flat-open closure or flat MH margins with bare RPE configuration with unsatisfactory visual prognosis despite closure [7–10]. The 5-Year Manchester Large Macular Hole Study found a higher success rate between 91 and 98% of surgical closure for large MHs with diameter in the range of 400–649 μm while MHs with diameter 650–1416 μm only achieved 76% [11]. Another study reported that the rate of MH closure was only 56% in eyes with a large MH of > 400 μm and about 10% of the closed MHs reopened after 6 months [12]. Moreover, surgical success in MH repair was found to be more than 90% within one year after the onset of the symptoms and lowered to 47.4% after 1 year [13]. Minimal visual gain can still be achieved after closing chronic MHs although this is associated with the MH duration [14].

Meanwhile, highly myopic MHs have been identified as another risk factor for failed MH repair, with at least 26 mm of axial length (AL) or refraction more than − 6 Diopter in spherical equivalent [15]. Anatomical success in myopic MH repair declined with the increasing AL, from 91.7% (AL: 26–29.9 mm) to 0% in eyes with AL > 30 mm [16]. The rigid ILM relatively resisted the anterior–posterior traction from the presence of posterior staphyloma and increasing AL and contributed to the formation of myopic MH [17]. Retinoschisis can also be seen commonly in persistent MHs and may contribute to late retinal detachment (RD) in unsuccessful surgical repair [18]. Other mechanisms that have been reported to cause surgical failure include residual tractional force from epiretinal membranes (ERMs), non-compliance with prone posturing, suboptimal intraocular tamponade, and postoperative cystoid macular edema [19–23].

There has been an increasing advent of more advanced and complex surgical techniques to treat these subsets of MHs with less favorable outcomes including persistent and recurrent MHs, high myopia with MH with and without RD, large MHs > 650 μm, and chronic MHs [24]. Therefore, this article aims to comprehensively review the available techniques to tackle these challenging MHs.

### Types of MH closure

It is also essential to identify the closure patterns of full-thickness macular holes (FTMHs) as there has been a correlation between the postoperative MH morphological features and the visual prognosis. An MH configuration with Type 2 or W-type has a less favorable visual outcome even though the MH is practically closed [10, 25]. Hillenkamp et al. also described refractory FTMH with subretinal fluid cuff as more likely to achieve anatomical success and improvement of best-corrected visual acuity (BCVA) following reoperation [26] (Table 1).

### Mechanism of MH repair

The early work in closing MHs was documented in the early 1990s, which included pars plana vitrectomy (PPV), removing adherent cortical vitreous, peeling of ERM, and a total gas–fluid exchange with long-acting intraocular gas tamponade. The goals were to relieve the anterior–posterior and tangential tractions on the vitreomacular interface and to appose the MH edges closer with gas endotamponade [22]. Surgical reattachment of detached macular could potentially improve the patient’s visual acuity as seen in 73% of the functional success rate postoperatively in 30 MHs that were closed [22].

With the advent of modern imaging modalities, the pioneering initiative by Kelly et al. has been modified into more sophisticated techniques to treat refractory MHs including ILM peeling, inverted ILM (i-ILM), i-ILM flap, pedicle ILM flap, retracting ILM door, autologous free ILM flap, non-ILM grafts, human amniotic membrane, hAM graft, adjuvant chorioretinal adhesives, and experimental mesenchymal stem cells (MSCs). Other methods that have been investigated were relaxing arcuate retinotomy, subretinal infusion, and hydrodissection.

Gliosis can be induced by peeling the ILM alone by activation of Müller cell fragments in the ILM. Thus by inverting the ILM flap over the MH, it acts as a scaffold for Müller cell tissue proliferation and may induce gliosis inside the retina and on the ILM surface, followed by MH filling [8]. Besides, an animal study had shown that Müller cells could produce photoreceptors following a toxic injury [27]. In addition, the ILM flap provides a barrier to prevent the vitreous fluid from entering the MH, thus allowing subretinal fluid reabsorption via the RPE [28]. In ILM peeling, the intraocular air or gas tamponade also provides the scaffold or creates a partition between the RPE and the fluid while enforcing further stabilization in the i-ILM flap [29].

The common practice for FTMH management is PPV with posterior hyaloid removal, ERM (if present) and ILM peeling, gas tamponade, and prone posturing postoperatively [30, 31]. Cheng et al. elucidated that peeling of ERM increases the rate of anatomical closure to 67% compared to 35% in the non-ERM peel group and the presence of excessive ERM postoperatively contributed to the reopening of MHs [32]. A meta-analysis in 2016 reported that ILM peeling was associated with a statistically significant reduction in the likelihood of MH reopening from 7.12% to 1.18% and significantly lower reoperation rates according to a large cross-sectional study [33, 34]. Another study demonstrated no significant difference between short-acting sulfur hexafluoride, SF_6_, and long-acting C_3_F_8_ in the hole closure rate, regardless of the hole size, stage, chronicity, or intraoperative dye used [35–37]. C_3_F_8_ may add benefit to the reoperation of persistent and recurrent MHs [36]. Facedown posturing offers potential superior visual acuity gain, although with no additional advantage in anatomical closure in large MHs [38].

### ILM peeling

The ILM peeling technique was first reported in a series of 39 eyes with FTMH with closure rate at 92% of eyes and 77% of closed MH displayed visual improvement at least two lines [39]. According to a Cochrane review and randomized controlled trials (RCTs), ILM peeling in idiopathic FTMHs confers superior benefits in increasing the likelihood of primary anatomical closure with additional benefits from dye-assisted peeling namely indocyanine green (ICG) and trypan blue (TB) [40, 41]. It lowers the risk of reoperation [5, 42], and extensive meta-analysis studies reported a better success rate in anatomical closure in the ILM peel group of 94.1–96% particularly in Stage III and IV and chronic MHs [43, 44]. However, there was no difference in the primary visual acuity improvement in peel and non-peel groups at six months [5], consistent with the findings of other trials [42, 45].

A long-term study has ascertained the safety of ILM peeling as the absence of late reopening of successfully closed MHs with significant improvement of the median BCVA from the baseline of 20/100 to 20/32 postoperatively. The authors also claimed that the visual acuity changes were independent of the duration of symptoms, the MH stages, or the number of operations [46].

Al Sabti and colleagues had successfully demonstrated closure of two extra-large MHs, measured at 1147 and 773 μm with extended ILM peeling up to the arcades and both eyes gained improvement in their postoperative visual function [47]. In MHs that failed to resolve after ILM peel with dye-assistance, enlarging the ILM-rhexis from prior peel may provide additional benefits. However, an early study on reoperation of persistent MHs that failed initial PPV showed a lower closure rate and poor visual prognosis despite closed MHs after secondary surgery [48]. Reoperation achieved varying closure rates from 46.7 to 68.9% in refractory MHs, which included enlarging ILM peel up to the vascular arcade and the posterior fundus to release further tangential traction on the MH [49, 50]. Meanwhile, metamorphopsia was alleviated with statistically significant improvement in visual acuity in closed MH cases, which was likely correlated to the alleviation of asymmetric elongation of foveal tissue [51].

ILM peel is associated with mechanical trauma to the retinal nerve fiber layer, including dissociated optic nerve fiber layer (DONFL) [52–54]. These changes appear on optical coherence tomography (OCT) as dark striae in areas of the previously peeled site in autofluorescence imaging and these were likely due to the damage to Müller cells from ILM peel that causes dehiscence of the optic nerve fiber bundles [55]. The formation of DONFL could also be a healing response following retinal nerve fiber layer (RNFL) dehiscence after ILM peel [19]. Although they potentially disappear within one to three months postoperatively, few authors had documented these changes in their cohorts from six up to 12 months with no definitive evidence of loss of visual function or microperimetry changes [19, 53, 55].

### i-ILM Flap

**Fig. 1:**
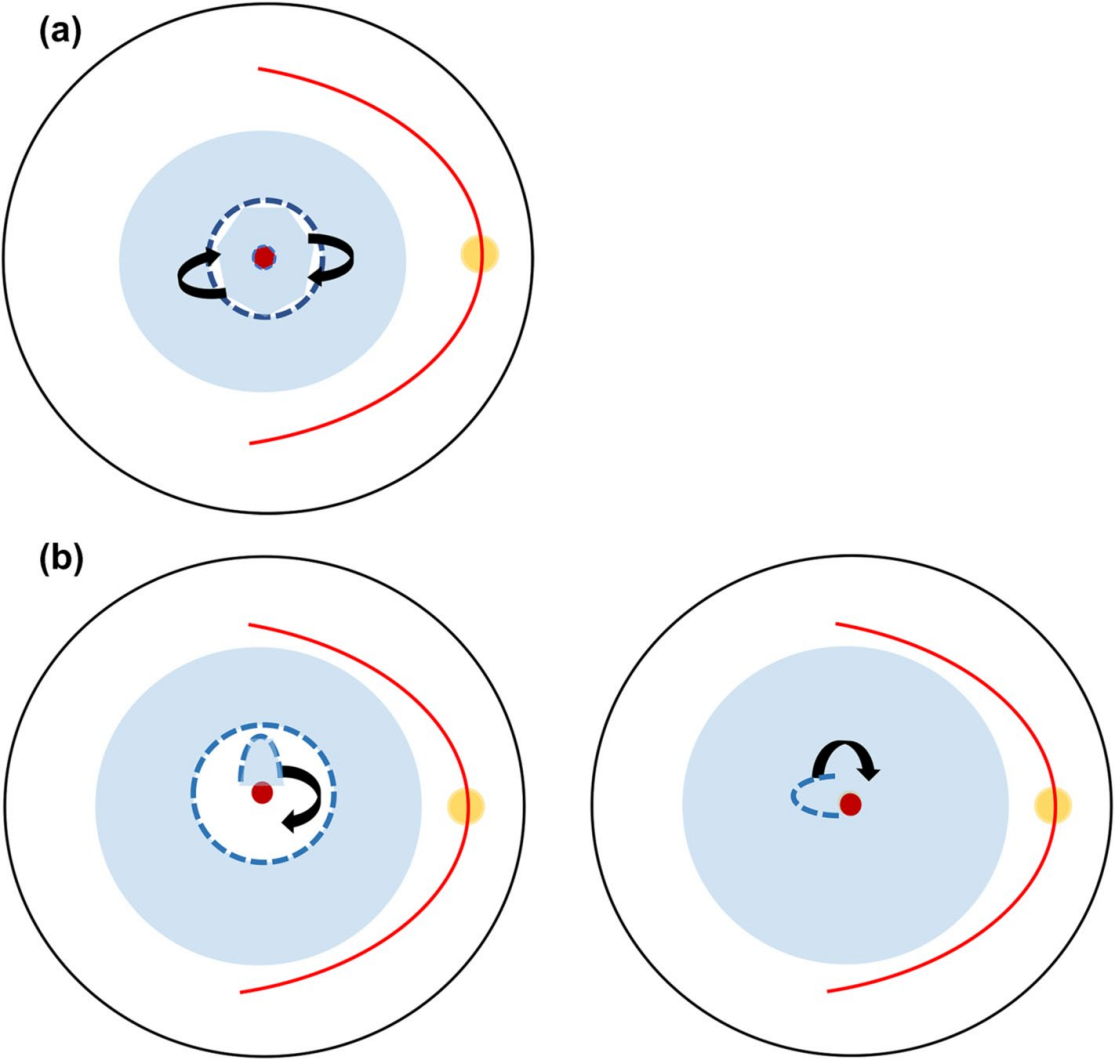
**a** i-ILM Flap by Michalewska et al. Following core vitrectomy, ILM was stained with TB and ERM was removed if present. Approximately 2 DD of ILM was peeled circumferentially and left attached to the edges of the MH. The peripheral ILM was trimmed and the central remnant of ILM was massaged until inverted over the MH. Subsequently, air–fluid exchange was performed with intraocular gas tamponade. Patients were advised to stay in a position that allowed them to see the air bubble in their central vision for 3–4 days.** b** Left—Shin et al. stained the ILM using brilliant blue-G (BBG) and removed the surrounding ILM but a 1 DD-sized flap superiorly to MH. PFC was injected over the flap for stabilization and repositioning if needed. Right—Michalewska et al. performed temporal i-ILM flap and about 2 DD of ILM was peeled temporally to the MH edge. The flap was then inverted over the MH until adequate coverage was obtained

Michalewska et al. performed PPV with i-ILM flap overlying MHs in large idiopathic MHs as per Fig. 1a and compared it to the conventional PPV with ILM peeling. The i-ILM flap group had 98% closure rates with a better functional outcome while the standard PPV group only achieved 88% anatomical success after the first surgery. A flat-open configuration was only seen about 2% in the former group compared to 19% in the latter [8]. Maneuvering the ILM flap intraoperatively imposes challenges to the surgeons as they face a steep learning curve to ensure effective surgical outcomes.

However, the original i-ILM flap method resulted in a multilayered membrane as identified in the postoperative imaging. Therefore, a single-layer inverted flap was introduced to create a more physiological and regular structure for gliosis and MH closure [56]. Figure 1b depicts variations of single-layer flaps by Shin et al. and Michalewska et al. [19, 57]. The single-layer i-ILM flap technique resulted in 83% closure with improved mean visual acuity [57]. Temporal i-ILM flap by Michalewska et al. achieved similar anatomical closure and visual improvement compared to the conventional i-ILM method for Stage IV MHs [19]. Another study reported that a higher success rate with the vertical flap was likely secondary to gravitational and more powerful inward tangential forces to close the MHs in a study that compared various sizes and locations of the i-ILM flap [21].

Few authors had incorporated adjuncts during surgery to stabilize and tamponade the flap in situ. Shin et al. used perfluoro-n-octane (PFC) before the fluid–gas exchange to prevent the displacement of the flap and for its repositioning if required [57], and others had included ocular viscoelastic device (OVD) and autologous blood clot [20, 58].

Minimizing the peeling area is also beneficial for reducing iatrogenic trauma in the papillomacular bundle area. DONFL was only observed localized to the peeled ILM region and these studies had proven that the large flap was not necessary for the i-ILM technique [19, 59]. Temporal i-ILM flap also caused less microvascular changes particularly in the deep capillary plexus and retinal sensitivity compared to ILM peeling [60, 61]. However, another study compared temporally versus nasally harvested i-ILM flaps in MHs < 600 μm and found significantly higher “deep inner retinal dimples” in the former group, 35% than the latter, and 5% with no correlation to the BCVA outcomes. Significant temporal macular thinning was greater in the temporal group and negatively correlated with BCVA. All eyes otherwise achieved 100% closure with 92% demonstrated U-type closure and ellipsoid zone (EZ) restoration was observed in 62% [58].

Folding i-ILM flap into MH also creates a multilayered appearance and it might hinder realignment of the outer retinal layers and thus vision improvement [62]. Few studies had recommended covering the MH with i-ILM flaps instead of “inserting” the flap into the MH due to potential trauma to the RPE in the fovea. An i-ILM flap covering MH showed significantly better BCVA, retinal sensitivity, and fixation stability compared to the “insert” group in idiopathic MHs repair [63]. The cover technique also yielded better postoperative visual gain as complete recoveries of the EZ and external limiting membrane (ELM) defects were seen even though both insert and cover groups achieved similar anatomical success [64]. Furthermore, Park et al. recommended i-ILM for superior visual acuity gain as no complete resolution of EZ and ELM defects was observed in the ILM insertion group in closing large MHs > 500 µm while i-ILM flap yielded better recovery of layers of photoreceptors [65].

Rizzo et al. analyzed 620 eyes with idiopathic or myopic MHs and found that vitrectomy with an i-ILM flap yielded statistically significant anatomical closure and improvement in BCVA compared to standard PPV with the ILM peel group. The anatomical closure rate in large FTMH was 96% versus 79% and in highly myopic MHs, it was 88% versus 39% between the i-ILM and ILM peel group. Overall, the i-ILM group had significantly better mean postoperative BCVA, 0.43 logMAR than 0.52 logMAR in the ILM peel group [66]. A systematic review demonstrated that the i-ILM flap method even achieved significantly better anatomical and functional success than ICG-assisted ILM peeling [67]. Meanwhile, the evidence for larger MHs was contradicting as Manasa et al. observed that i-ILM is more superior anatomically and functionally to ILM peel in MHs > 600 µm [68] but additional trials did not demonstrate any difference between both surgical groups in these larger MHs [69, 70]. Another study did not find any difference in anatomical closure between the standard ILM peel and i-ILM flap techniques in medium-large (400–500 µm) and extra-large MHs (> 550 µm) but the authors recommended that the i-ILM flap is more effective in extra-large MHs as the group yielded 100% anatomical closure postoperatively compared to ILM peeling [9].

In myopic MHs with RD, an i-ILM flap is associated with significantly better MH closure and retinal reattachment compared to the ILM peel group although there was no difference in the postoperative BCVA in both groups. It reduced the risk of recurrent RD as unclosed MHs may increase the risk of retinal re-detachment [71]. All myopic MHs with RD had successful retinal reattachment in both ILM peel and i-ILM flap groups but significantly better recovery rates of the ELM and EZ layers were observed in the latter group [72]. The i-ILM flap technique also closed all myopic MH without RD and enhanced foveal architectural regeneration as seen within 12 months postoperatively with an associated mean visual acuity gain of 0.64 logMAR [73]. A meta-analysis demonstrated that i-ILM flap has a significantly higher and more superior anatomical closure rate in each subgroup of MHs, large idiopathic MHs and myopic MHs with or without RD [74].

Long-term analysis showed mixed evidence of the efficacy and safety of i-ILM. The visual recovery in the i-ILM flap group was significantly higher at 3 months compared to ILM peeling but no difference was identified at a longer follow-up more than 6 months while performing better anatomical success than the ILM peel group [75]. Another study had shown a promising impact of i-ILM flap in treating large MHs as the recovery of the ELM had been identified as early as 3 months and yielded significantly higher foveal restoration than the ILM peel group [76]. Nevertheless, there is a strong correlation between the BCVA and the integrity of the EZ layer, independent of the presence of the ILM flap [77]. However, the i-ILM flap group showed lower recovery rates of the EZ and ELM and required a longer recovery period of the ELM postoperatively than the ILM peel group. Thus, the changes in BCVA gain are significantly smaller in the i-ILM group, and the authors suggested that there is a limited role of i-ILM flap in managing refractory MHs [78]. A recent publication studied the changes of temporal i-ILM flap at 1 month and 6 months postoperatively and found that ILM flap contracted significantly especially in the younger cohort and one eye had more than 20% shrinkage and required reoperation [79].

### SWIFT

**Fig. 2:**
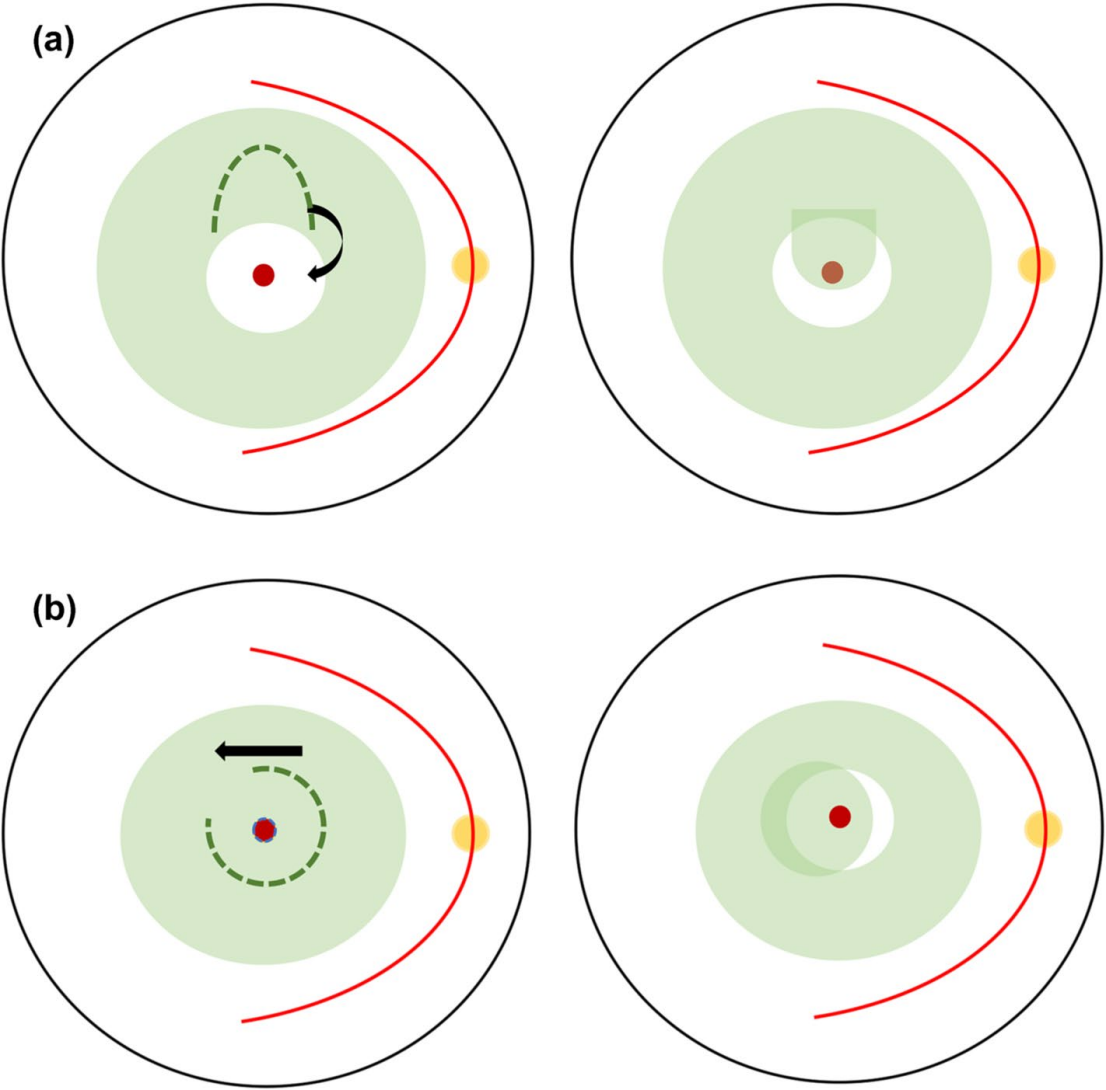
**a** Tabandeh et al. performed a distal superior ILM flap from the MH and transposed it over the MH.** b** Formation of pedicle ILM by Hu et al. PFCL was injected to protect the MH and the exposed RPE before staining the ILM with ICG or BBG. Then the ILM peeling was performed circumferentially around the MH for at least 2 DD and left attached to the superior temporal retina. The pedicle ILM then rotated and transposed over the MH, with its nasal part fully covered the MH while stabilised and flattened under a larger bubble of PFCL followed by air-fluid-PFCL exchange. The authors chose either SO or autologous whole blood with C3F8 gas to prevent displacement of the ILM pedicle transposition and patients were advised to prone-posturing for 3 days

In Fig. 2a, Tabandeh et al. modified i-ILM flap to SWIFT or “superior wide-base internal limiting membrane flap transposition” for complicated MHs including refractory, chronic, and highly myopic MHs with previous ILM removal. In the study, 17 complicated MHs were included in the retrospective series with a mean basal diameter (MBD) of 899.4 μm and the SWIFT method yielded 94% closed MHs and improved mean VA from baseline 0.88 to 0.54 logMAR at the final visit. By using the ICG fluorescence imaging, the ICG-stained SWIFT flap was visualized postoperatively and 82% MHs had full coverage of the flap, whereas the remaining MHs had partial or no coverage. However, one MH was closed in eyes that were not completely covered by the flap [80].

Tabandeh highlighted that the cross-sectional view of OCT may not be able to distinguish the flap’s location and its status. The author described that the “en-face” visualization of ICG-stained SWIFT flap using confocal laser imaging at 795 nm had shown variable degrees of ICG hyper-fluorescence, which indicated to the position of the flap and the MH coverage, whereas the hypo-fluorescence area reflected the flap harvest site. As the ICG signal may fade, the author recommended ICG imaging between two and four months, postoperatively, after the resolution of gas endotamponade [81].

### Pedicle ILM transposition

Hu et al. argued that a single-layer and non-inverted flap is more physiological than the i-ILM flap technique as theoretically the glial proliferation and the macular closure are more favorable if the retinal side of the flap covers the MH as per Fig. 2b. The method achieved 91.7% of V-type closure in large MHs and the closure was observed as early as day 1 postoperatively in six SO-filled eyes. The mean BCVA was significantly improved from baseline at 1.23 logMAR to 0.67 logMAR postoperatively and retinal sensitivity and multifocal electroretinogram (mERG) responses were significantly improved in correlation to attached ILM plug on the macular and restoration of the retinal layers [82].

A preliminary study demonstrated the pedicle ILM transposition as a primary method in successfully closing three very large MHs with a minimum diameter > 700 μm out of four eyes. One eye achieved U-type with one line BCVA improvement and two eyes yielded V-type closure with 1–2 lines of BCVA gain [83]. While the authors argued that the technique is not feasible in eyes that had undergone prior ILM peel, the pedicle ILM transposition method had been used in two refractory MHs when there was no ILM available around the MHs. The pedicle was created by peeling the remaining inferior ILM toward superiorly, which then transposed over the MH while still attached to the superior margin of MH. The method eliminates the risk of flap loss observed in autologous free ILM transplantation for managing recurrent and persistent MHs [84]. However, some authors reported that the flap contracted over time and reopened the MH [85].

### Retracting ILM door

**Fig. 3:**
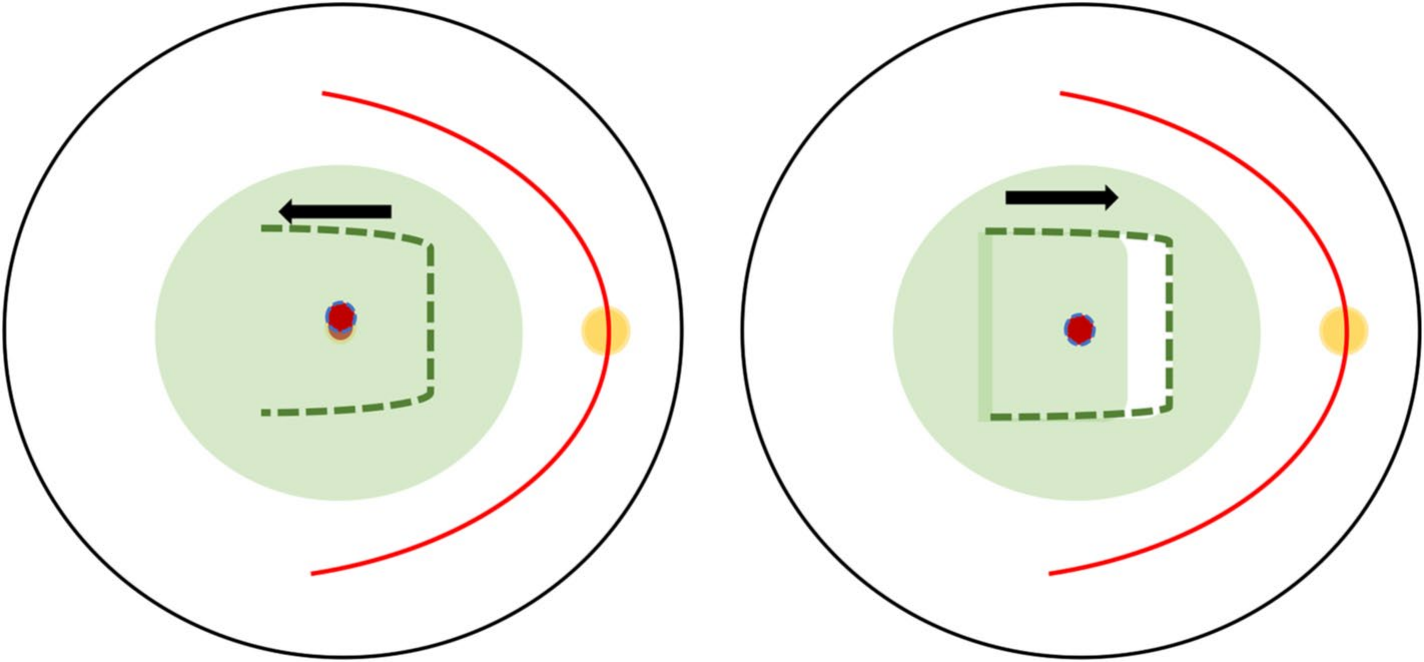
ILM retracting door. The ILM was stained using indocyanine green (ICG) and a large flap was created starting nasally to temporally, including over the fovea and the MH area. The flap which now hinged temporally then freely draped over the MH and thus the nasal ILM flap covered the MH

A novel technique was introduced that successfully treated two myopic MH with and without RD by creating a hinged retracting ILM flap as per Fig. 3. This is to relax the rigid ILM seen in highly myopic eyes and the retracting flap over the MH will provide the scaffold needed for the proliferation of cells and the migration of photoreceptors. Both eyes gained remarkable visual gain from counting fingers and 20/80 to 20/50 postoperatively. This technique also eliminated excessive manipulation and prevented the displacement of ILM flap as well as preserving ILM unlike the i-ILM flap, which could be potentially beneficial in cases with thin retinae and for future use if required [17]. This method also maintains the original orientation of ILM and Müller cells although its long-term profile remains unknown.

### Autologous ILM free flap

Surgical failure in MHs that already had ILM peel somehow may limit further potential treatments in the refractory MHs. Morizane et al. introduced autologous transplantation of the ILM in cases with refractory MH that failed to close after previous ILM removal. The authors harvested a similar diameter of ILM flap to the MH and during the intraoperative period, the infusion was turned off to prevent the turbulence from displacing the free flap, and then an OVD was injected overlying the flap and MH. Even in complicated cases including traumatic and myopic MHs, the technique achieved MH closure in 90%, and 80% had improvement in visual acuity by more than the logarithm of the minimum angle of resolution, logMAR 0.20 [86].

Similarly, autologous ILM free flap yielded 93.3% closure in recurrent primary MHs with a significantly better functional outcome at 3 months than the control ILM enlargement peel group. The former group also achieved lesser defects of the inner and outer retinal segments [87]. Multiple free ILM flaps and double ILM layered insertion into highly myopic MH with RD also achieved superior closure rate and assisted retinal reattachment in all cases versus ILM peeling alone [88, 89]. An imaging study found that the autologous ILM free flap promoted prolonged glial proliferation that led to the closure of large or refractory MHs with evidence of foveal depigmentation and fibroplasia and partial regeneration of EZ and ELM defects. These foveal changes however did not affect the visual outcome but further study is needed to elucidate the safety profile of this technique [90].

Managing the autologous ILM free flap intraoperatively can be challenging as poor visual of the flap despite adequate dye staining. Dai et al. injected the OVD into the MH to gently lift the margins around the hole thus allowing the free large flap to be placed and fixed under the MH edges [91]. Other studies had incorporated PFC to harvest and stabilize autologous ILM intraoperatively with relative success in anatomical and visual outcomes [56, 92]. The authors also cautioned the risk of retinal injury as the flap may get stuck and difficult to dislodge from the forceps and the free ILM flap’s orientation can be difficult to be identified intraoperatively [56].

Besides the difficulty in repositioning the free ILM flap, surgeons may find it challenging to harvest the right size for the graft to avoid redundancy or inadequate coverage of the MH. Lowering the infusion pressure during transplantation, down to 10 mmHg, can assist in securing the free flap inside the MH to prevent excessive movements from the turbulence [93]. Other authors recommended performing the gas–fluid exchange at the surface of fluid level with passive aspirations toward the end to prevent the flap displacement. Fung et al. demonstrated that transposition and tucking autologous ILM without stabilization aids yielded an 87.5% MH closure rate with a significant mean VA gain of 1.13 lines in cases with refractory MHs although it harbored risk of inflicting trauma to the RPE [94].

### Autologous lens capsular flap transplantation (LCFT)

If autologous ILM free flap is not readily available due to prior wider ILM removal or difficulty in obtaining the fragile peripheral ILM, Chen et al. proposed transplantation of lens capsular flaps to close refractory MHs. Lens capsule has a higher density than ILM; thus, manipulating it during surgery is more convenient as it gravitationally settles down over the MHs once released from micro-forceps. There was no statistically significant difference in the visual acuity improvement in anterior and posterior capsule groups, but a better anatomical closure rate was seen in the former group as the anterior flap was more rigid than the posterior flap. The concomitant cataract surgery might affect the actual visual acuity outcome of the anterior group. Nonetheless, LCFT positively impacts the visual function as visual improvement was also seen in the posterior capsule group without the cataract surgery [95].

Authors of a long-term study with a mean follow-up of 18.5 months demonstrated that LCFT is ideal as a first-line treatment for refractory MHs. The study investigated LCFT either autologous or allogenic in origin in 48 MHs with a mean diameter of 1102 μm. In this study, 96% of MHs were closed with significantly improved median visual acuity from baseline 1.79 logMAR to postoperative 1.00 logMAR. The authors also recommended adjunct whole blood to tamponade the LCFT to prevent its displacement [96].

### Autologous neurosensory retinal transplant (ART)

Harvesting and transplanting the free ILM flap can be challenging in myopic MHs due to poor staining of the ILM and fragile nature of the eye from weakened retinal-posterior pole adherence, which is secondary to RPE and choroidal atrophy and posterior staphyloma [86, 97]. Grewal et al. demonstrated the successful use of neurosensory retinal graft to close refractory myopic MHs with RD in a complex ocular case as autologous lens capsule and ILM free flaps were unattainable for transplantation. A 2 DD ART graft was harvested from the neurosensory retinal site superior to the superotemporal arcade, and the graft site was secured by endolaser and endo-diathermy. The graft was stabilized in situ by perfluoro-*n*-Octane heavy liquid (PFCL) followed by direct PFCL–silicone oil exchange. MH was observed closed at 1-week with gradual improvement in visual acuity, visual distortion, and scotoma size. These improvements were also supported by an increase in retinal sensitivity from 7.5 dB to 12.3 dB at 3 months while the graft site showed no evidence of ERM and RD. The authors reiterated that i-ILM flap is not indicated in refractory MHs that had failed primary ILM peel and PPV [98].

A multicenter retrospective study showed that 88% of 41 refractory MHs with an MBD of 1468.1 ± 656.4 μm were closed with ART and 52.3% of the closed MHs showed visual improvement [99]. Other case reports demonstrated successful MH closure following ART in persistent FTMH and complicated MH in recurrent myopic RD with visual acuity gain from BCVA pre-operative 20/800 and light perception to postoperative 20/100 and 20/400, respectively [100, 101]. A recent study demonstrated ART closed 76.92% of refractory MHs with an MBD of 1615.38 ± 689.19 µm and at one year, six of the closed MHs achieved full restoration of the myoid/ellipsoid layer. However, there was no statistically significant visual gain observed, and one eye developed posterior vitreoretinopathy and ERM from the ART harvested site [102]. Although these changes did not affect the patient’s visual acuity, a long-term study is warranted to investigate ART’s safety profile as only transient graft edema was only reported in other studies [99, 100, 103].

ART showed anatomic integration, according to Grewal et al., as significantly reduced EZ and ELM defects were demonstrated on the OCT and integration of the graft within the adjoining retina and its migration to the MH [99]. A small series of four eyes had shown a better EZ and ELM defects recovery in cases whereby the grafts were placed under the edges of the MH compared to the epiretinally positioned grafts as its closer contact with the RPE promotes improved tissue integration and photoreceptor survival [104]. Tabandeh studied the graft’s vascular profile after large ART in two giant MHs with an MBD of 2914 μm. Like other older studies that had demonstrated the graft’s anatomical integration into the surrounding retina, Tabandeh also observed the graft’s vascular reperfusion on angiography imaging at the earliest 6 weeks. He argued that the ischemic 5 DD retinal graft stimulated adequate angiogenesis that promoted anastomosis between the graft and the retina. Subsequently, the angiogenic drive diminished as reperfusion took place and prevented exaggerated angiogenesis response. He also described various stages of the graft’s physiological changes in correlation to MH’s size and visual improvement [105].

Displacement of the neurosensory retinal flap can still occur during surgery and postoperatively. Thus, Grewal et al. recommended harvesting and maneuvering the graft under the PFCL, which can be left as a safe short-term tamponade for 1 to 2 weeks although it requires repeat surgery for its removal [99]. Other smaller studies had successfully used deuterium oxide, autologous blood clots, and viscoelastic to secure the retinal graft in situ in managing refractory MHs, including recurrent MHRD [103, 106, 107]. While positioning ILM or lens capsule flaps inside MHs are at risk of iatrogenic trauma, Grewal et al. justified that the ART minimizes the surgical trauma as it can be positioned over the MH due to its thicker and sturdier graft [99].

Overall, the neurosensory retinal free flap created a barrier between the vitreous and the subretinal space thus to let the subretinal fluid excretion by the RPE and to scaffold glial repair, though the exact mechanism of the graft’s structural integration is yet to be clarified [98, 103, 106]. However, ART may not be indicated if the retinal graft is not viable for harvesting including ischemic or inflamed retinae, neovascularization, or extensive scarring of the chorioretinae.

### Human amniotic membrane (hAM) transplantation

hAM has been utilized in ocular surface diseases and provides a biological scaffolding for conjunctival growth. A porcine study supported the finding of hAM in promoting the growth of RPE tissue in the subretinal space [108]. Rizzo et al. first demonstrated filling of the MHs with layers of the neurosensory retina in all eight eyes at one week following transplanted hAM into the subretinal space via the MH. All closed refractory MHs had improvement in the visual acuity from pre-operative 1.48 logMAR to 0.48 logMAR at six months. The authors also successfully used hAM to treat six retinal breaks in complex RD cases in this prospective series [109].

While in highly myopic MHs with AL > 30 mm, dislocated hAM plug may occur after intraocular gas reabsorption and require repeat procedure as seen in one eye from a series of 16 eyes, MHs closure was readily seen on OCT examination in 15 eyes at two weeks. Although a slight improvement was observed in their visual acuities postoperatively, the 100% anatomical closure in all eyes remains a success given a potential complicating RD in persistent MHs in myopic eyes [110]. Both studies demonstrated that hAM plugs successfully induce growth of retina into organized layers that leads to recovery of original retinal function virtually [109, 110].

A meta-analysis demonstrated that the hAM-treated group achieved at least double the visual gain compared to other reported techniques for refractory MHs. This is likely attributed to the placement of the hAM compared to other tissue grafts as it was transplanted under the margins of the FTMHs and in the subretinal space. The close contact between the RPE and the graft protected the MH from vitreous and also promoted RPE proliferation and retinal restoration as per in vitro studies [108, 111]. Nevertheless, the anatomical success rate between hAM and other autologous ILM and non-ILM grafts transplants was similar to close refractory MHs [112].

### Others

Multiple studies had investigated subretinal infusion, MH hydrodissection, and relaxing retinotomies to close refractory MHs [112–116]. The goals are to increase retinal tissue compliance and to mobilize the edges of the MHs, thus improving the likelihood of the refractory MHs to close and attain improved visual outcomes. The outpatient fluid–gas exchange has been studied by additional intraocular gas top-up and better results were achieved with the long-acting gas compared to the short-acting gas [117]. It was more feasible, cost effective, and tolerable for patients. Heavy silicone oil like Densiron 68 had been used in the retreatment of persistent and recurrent MHs and it yielded better surgical outcomes compared to gas and silicone oil endotamponade [118]. It also has a better safety profile than silicone oil [30]. The experimental use of MSCs in treating large and recalcitrant MHs was studied by Zhang et al. with promising results although a larger sample size and long-term study are warranted [119].

## Conclusion


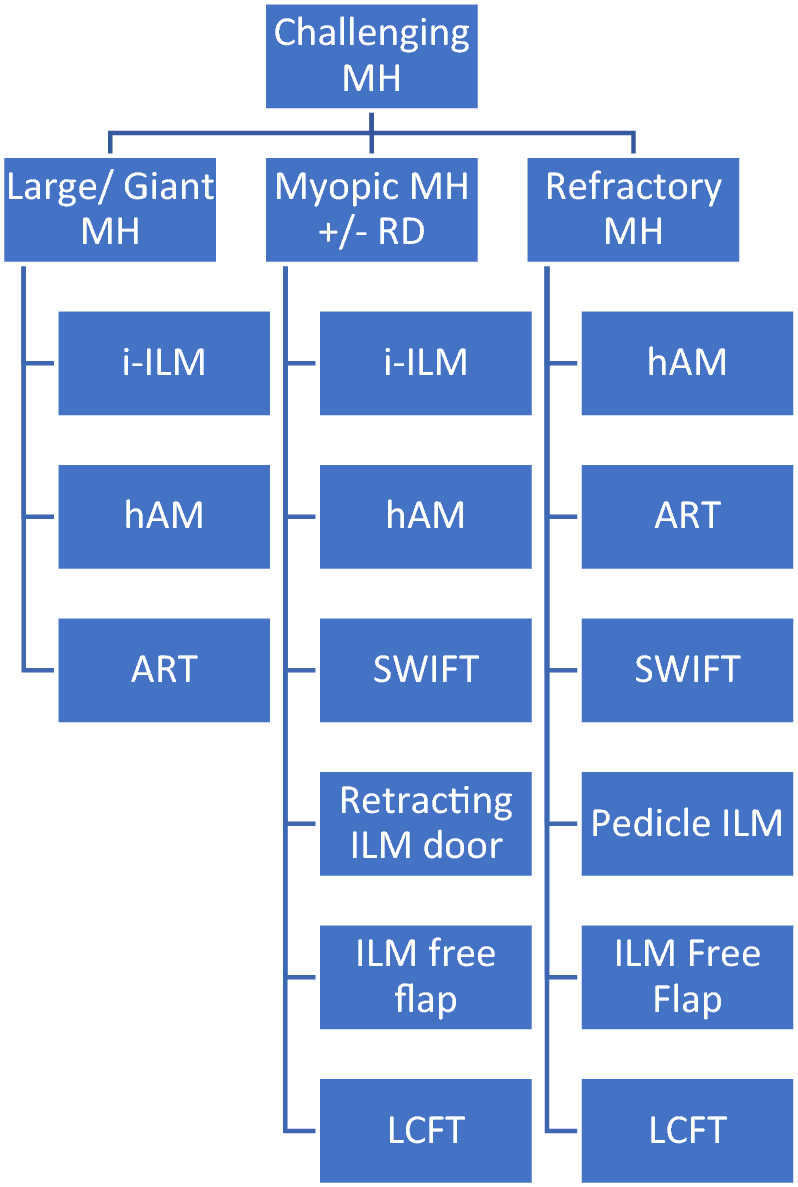


As all MHs do not happen equally, there is no definitive method or technique to propose the best method to close difficult MHs. There is extensive evidence supporting the i-ILM flap as the primary treatment for large idiopathic MHs and highly myopic MHs. With the growing data on the experimental use of various surgical techniques and discoveries of novel adjuvants that exert additional biological advantages on top of the scaffolding to close the MHs, it is exciting to see the vast alternatives besides i-ILM flap to treat the devastating disease. There is a shift of trend from ILM peeling to ILM flap manipulation to preserving as much ILM for future utilization if required.

The patient’s wellbeing must be factored-in in considering if it is worthwhile to operate on challenging MH with poor visual baseline or even reoperating in MHs with unfavorable outcomes. Patients will be subjected to a stricter ordeal of postoperative posturing and it costs them more time, expenditure, and effort. However, it can be argued that there is a very limited window to stabilize vision or to prevent further deterioration of macular thus there may be a benefit to surgery, even if the functional outcome is not expected to improve greatly. An opened refractory myopic MH imposes the risk of recurrent RD, which can lead to devastating sequelae if left untreated.

The end goals of MH surgery are to remove or mitigate the forces that “open” the MH, to bridge or to provide scaffolds to join the edges of the MH together, thereby closing the MH, in the hope of improving vision. Visual success is not proportionally commensurate with anatomical success as visual gain seen in extra-large and chronic MHs was deemed inferior even after successful closure, highlighting the importance of determining the pre-operative size of the MH as one of the predictive factors in the determination of visual benefit postoperatively. The expanding niche in improvising and inventing surgical techniques with more novel adjuvants will likely improve the surgical outcomes even in MHs with the worst prognosis.

Overall, most studies have similar limitations, including selection bias and small sample size, and they require longer follow-up times to elucidate any potential Type II errors. Furthermore, all the above techniques that were collectively discussed may not find unanimous support of all the retinal surgeons worldwide, as different surgeons may find specific techniques work better in their care and that patient’s selection and postoperative expectations may also be the deciding factors in choosing the preferred approach in the management of these challenging MHs.

**Table 1:** Different types of configurations of MH closure

Authors	Imai et al. [10]	Kang et al. [25]
Closure patterns of FTMH	U-type: normal foveal contour	Type 1: closed MH without foveal defect of the neurosensory retina
	V- type: Steep foveal contour	
	W-type: Foveal defect of the neurosensory retina	Type 2: a persisting foveal defect of the neurosensory retina postoperatively although the rim of the MH is attached to the RPE and the cuff is flattened

## Data Availability

Not applicable.

## Abbreviations

MHs: Macular holes
FTMHs: Full-thickness macular holes
ILM: Internal limiting membrane
RPE: Retinal pigment epithelium
PVD: Posterior vitreous detachment
ERM: Epiretinal membrane
RD: Retinal detachment
EZ: Ellipsoid zone
ELM: External limiting membrane
PPV: Pars plana vitrectomy
RCTs: Randomized controlled trials
ICG: Indocyanine green
TB: Trypan blue
BBG: Brilliant blue-G
DD: Disc diameter
DONFL: Dissociated optic nerve fiber layer
BCVA: Best-corrected visual acuity
Log MAR: Logarithm of the minimum angle of resolution
hAM: Human amniotic membrane
APC: Autologous platelet concentrate
OCT: Optical coherence tomography
TGF-β_2_: Transforming growth factor beta 2
SO: Silicone oil
